# Cross sectional study to determine chloroquine resistance among *Plasmodium falciparum* clinical isolates from Khartoum, Sudan

**DOI:** 10.12688/f1000research.13273.1

**Published:** 2018-02-20

**Authors:** Walaa Salah Abdulla Mohammed, Kyakonye Yasin, N.S. Mahgoub, Muzamil Mahdi Abdel Hamid

**Affiliations:** 1 Faculty of Pharmacy, Sudan International University, Khartoum, Sudan; 2 Faculty of Pharmacy, International University of Africa, Khartoum, Sudan; 3 Department of Medical Laboratory Sciences, Faculty of Medicine, Sinnar University, Sinnar, Sudan; 4 Department of Parasitology and Medical Entomology, Nile College, Khartoum, Sudan; 5 Department of Molecular Biology, National University Research Institute (NURI), National University, Khartoum, Sudan; 6 Department of Parasitology and Medical Entomology, Institute of Endemic Diseases, University of Khartoum, Khartoum, Sudan

**Keywords:** Chloroquine, resistant, sensitive, P. falciparum, pfcrt

## Abstract

**Background:**  Malaria continues to present a global health threat; the World Health Organization (WHO) reported 214 million cases of malaria by the year 2015 with a death rate of 438000. Sudan is endemic to malaria with over 95% of malaria cases due to *Plasmodium falciparum*. Chloroquine is a well-established drug in the treatment of *P. falciparum* malaria although its use has declined since its introduction as the drug of choice in treatment of malaria in Sudan. The mechanism of resistance has been attributed to mutations in *P. falciparum* Chloroquine resistance transporter gene coding for a key food vacuole proteins. In current study we aimed at verifying the genetic cause of resistance to Chloroquine in field isolates of *P. falciparum*.

**Methods:** Twenty *P. falciparum* cases were diagnosed from East Nile hospital in Khartoum and recruited in the investigation. Nested PCR was conducted to isolate mutation region in the PfCRT gene and the amplicons were sequenced using Sanger sequencing technique (Macrogen, Soule Korea).

**Results:** 16/20 (80%) of the field isolates contained base pair mutation of codon 76 in the *pfcrt *gene thus being resistant to chloroquine treatment and only 4/20 (20%) did not contain such mutation.

**Conclusions:** High treatment failures associated with Chloroquine treatment is evident of the high prevalence of mutant strains of *P. falciparum* field isolates thus suggesting the reduced relevance of Chloroquine as a treatment choice in the management of *P. falciparum* malaria.

## Introduction

The use of chloroquine in low middle income countries (LMICs) has helped to reduce mortality and morbidity. In 1940, sixteen years after its discovery, Chloroquine had been used as a first drug of choice for malaria treatment due to its high efficacy, especially in highly endemic areas in Africa ^1^. It has been used as the main treatment for malaria in Africa for over 50 years. Since the late 1980s, resistance to chloroquine has rapidly increased across much of Eastern and Southern Africa ^2^.

Drug resistance of *Plasmodium falciparum* represents one of the greatest public health challenges in Africa. Tropical Africa accounts for more than 90% of the 300–500 million clinical cases of malaria occurring each year ^3^. Malaria is transmitted by the female anopheles mosquito with *P. falciparum* being most prevalent; however recent studies in most parts Sudan have indicated a high prevalence of *P. vivax* infection ^4^.

Chloroquine resistance is associated with a parasitic digestive vacuole, in which the toxic free heme is converted into an insoluble non-toxic crystalline form called hemozoin ^5^. In sensitive parasites, Chloroquine diffuses across the food vacuole membrane and accumulates inside it to inhibit hemozoin crystallization, which leads to formation of another highly toxic-dimeric hematin complex ^1, 5^. Resistant strains fail to accumulate sufficient Chloroquine inside their food vacuoles as a result of mutations in the FV membrane proteins ^5^. Mutation in *P. falciparum* Chloroquine resistance transporter gene *Pfcrt*, which codes for one of the food vacuole proteins is considered the strongest predictor for Chloroquine resistance ^5^. This mutation leads to the substitution of the amino acid lysine with threonine at position 76 (K76T) ^5^.

By 2004, the National Malaria Control Programme of Sudan updated the policy of malaria treatment due to high prevalence of Chloroquine resistant cases. In this policy, Artesunate + Sulfadoxine-Pyrimethamine (AS + SP) was adopted as the first-line treatment for uncomplicated *P. falciparum* malaria, and Artemether-Lumefantrine (AL) was adopted as a second-line treatment ^6^.

However, until now, Chloroquine is still considered as one of the cheapest and safest drugs ever used for malaria treatment. Besides, recent clinical observations show a decreasing trend in prevalence of *P. falciparum* Chloroquine resistance, which has brought Chloroquine reintroduction back into the discussion about the management of malaria ^7, 8^.

Hence, in this pilot study, we aimed to assess the prevalence of Chloroquine resistant *P. falciparum* from clinically isolated samples by sequencing the *Pfcrt* gene in patients with uncomplicated malaria in Khartoum, Sudan.

## Methods

### Ethical statement

This study was reviewed and approved by Institute Of Endemic Diseases Research and Ethics Committee (ethical approval number 6/2016). Oral informed consent was obtained from participating patients or from parents or guardians for the case of minors (under 19 years). Oral consent was obtained over written consent, since the majority of the patients included in this study were illiterate.

### Study site and patients

A cross sectional study was conducted between December 2015 and January 2016 within Khartoum East Nile Hospital. According to Isaac and Michael (1995) suggestion of pilot study sample size, twenty patients, between age 4–55 years, have been randomly selected from the hospital attendance sheet ^9^. After getting positive results of uncomplicated malaria diagnostic tests, the selected patients were immediately asked to be a part of the present study. Thirteen of the patients were women and seven were men. Demographic data was obtained from participating patients including ethnicity and recent history of malaria infection. Only patients confirmed with *P. falciparum* monoinfection were included in further studies. Pregnant or lactating women and patients presenting with signs or symptoms of severe malaria or having a history of taking antimalarials during the previous month or another febrile disease requiring treatment were excluded.

### Sampled and malaria microscopy

1ml blood sample was collected from each patient and stored in a labeled EDTA tube, thick and thin blood films were prepared for microscopical examination to detect and isolate *Plasmodium* species and estimate of parasitemia level.

### Parasite DNA extraction

1ml blood sample, for mutation molecular detection, was collected from each patient and stored in a labeled EDTA tube. A mixture of 1ml of Saponin (0.5%) (Sigma-Aldrich) and 150 µL of each blood sample was incubated overnight at 4°C, and then centrifuged at 6000 rpm speed. 1ml of phosphate-buffered saline (PBS) (Sigma-Aldrich) was added and the mixture was centrifuged, at 6000 speed, for four consecutive times. 70 µL of chelex (Bio-Rad) and 30 µL of water were added to the precipitant and the mixture heated at 95°C for 20 minutes in 4 rounds (5 min/round). After every round the mixture was vortexed for 1 minute. DNA was collected and stored at -20°C until a PCR reaction was conducted.

### Molecular detection of Pfcrt K76T mutation

For round 1 of nested PCR, 2µL of extracted DNA was multiplied using 0.5µL of Taq DNA polymerase, 2µL buffer, 2µL dNTPs, 10.5µL nuclease-free PCR water, 1.5µL of forward primer (CRTP1; Macrogen), and 1.5µL reverse primer (CRTP2; Macrogen). Primer details are provided in Table 1. The PCR initial denaturation was conducted at 95°C for 3min, 94°C for 30 s for the successive denaturation steps, DNA annealing was set at 56°C for 30 s and elongation at 60°C for 1 min, a total of 45 cycles were conducted and final extension at 72°C for 5min.

**Table 1.** Primers used in the present study.

Primer	Sequence	Product size
Outer forward primer CRTP1	5’CCGTTAATAATAAATACACGCAG3’	537 bp
Outer reverse primer CRTP2	5’CGGATGTTACAAAACTATAGTTACC3’	
Nested forward primer CRTD1	5’TGTGCTCATGTGTTTAAACTT3’	145 bp
Nested reverse primer CRTD2	5’CAAAACTATAGTTACCAATTTTG3’	

For round 2 of nested PCR, 1 µL of round 1 product was used with CRTD1 (Macrogen), as forward primer and CRTD2 (Macrogen) as reverse primer, with the PCR profile as summarized below.

Initial denaturation at 95°C for 5 min and 92°C for 30 s for succeeding denaturation steps, annealing was conducted at 48°C for 30 s, elongation at 65°C for 30 s. Final elongation was conducted at 65°C for 3 min. The DNA amplicons were run in 1.5% agarose gel stained with 2% ethidium bromide electrophoresis gel and finally bands were visualized under UV light. The amplicons were then sequenced using Sanger sequencing techniques at Macrogen (Soul Korea). All sequences were obtained and compared to the nucleotide sequence of *Plasmodium falciparum* 3D7 reference strain provided by Biomedical Primate Research Centre (BPRC, Netherlands).

### Bioinformatics analysis

Sequence cleaning and blast analysis were conducted using FinchTV software version 1.4.0. Visualization of nucleotide sequences and alignment with wild type sequences was conducted using Bioedit software version 7.2.5.

## Results

**Figure 1.**
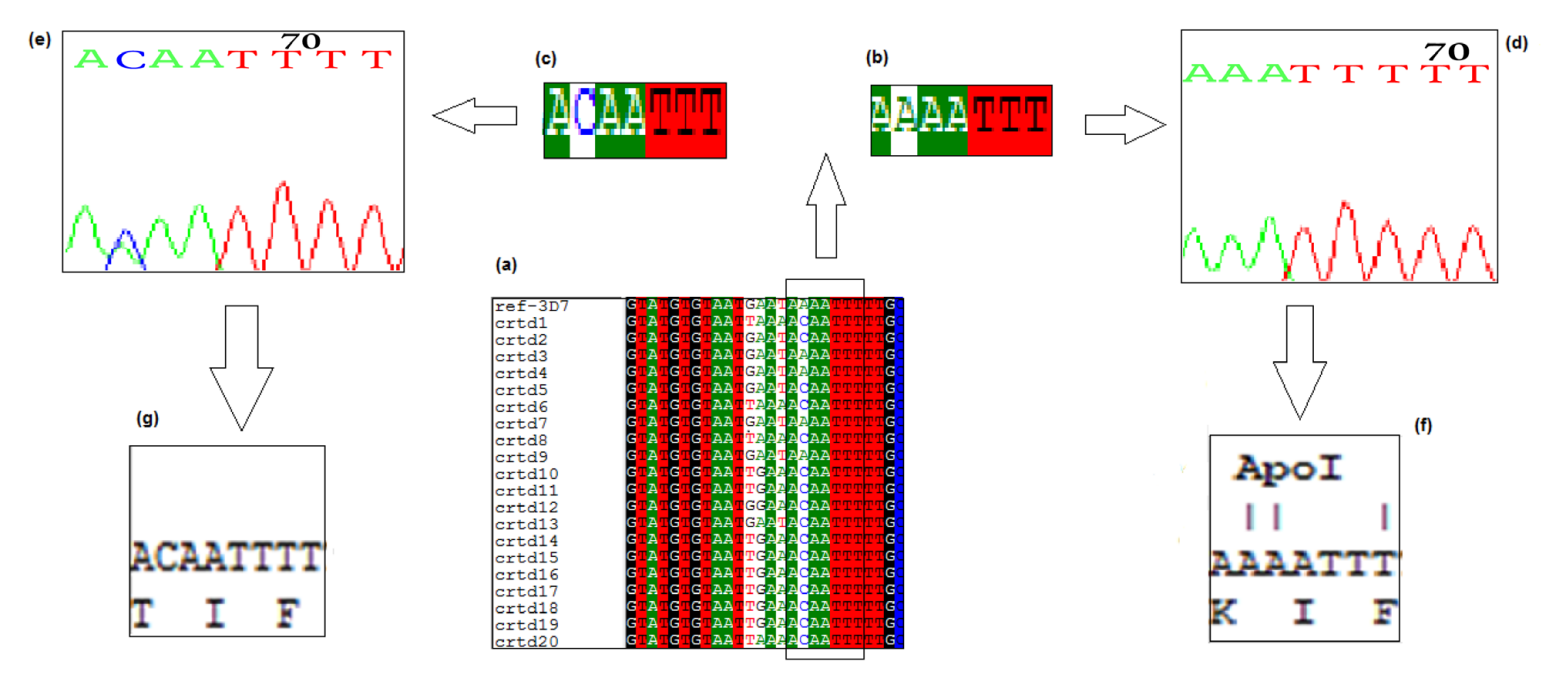
( **a**) Multiple sequence alignment highlighting the *Pfcrt* gene at the Apol 1 cutting region. ( **b** and **c**) Highlight point mutation in *Pfcrt* gene where the A nucleotide in a wild-type is substituted with C nucleotide in the mutant strain, ( **d** and **e**) the nucleotide sequence chromatogram of wild type and mutant strains respectively. ( **f** and **g**) The translated amino acid in wild and mutant strains, respectively.

All samples from 20 patients were successfully amplified. Nucleotide sequences were obtained and analyzed using bioinformatics analysis. Nucleotide sequences were aligned and compared to standard sequence of *Plasmodium falciparum* 3D7 reference strain. 16/20 (80%) of the sequenced DNA contained ACA triplet codon, which codes for Threonine at the Apol 1 restriction enzyme cutting region. Only 4/20 (20%) samples contained the AAA triple codon which codes for Lysin in the wild type *Pfcrt* gene, as shown in Figure 1.

Raw sequences for the 20 patients included in the present study Files included: sequence in pdf format, sequence for opening in FinchTV software (.ab1 file), and sequence for opening in Notepad (TXT file). Click here for additional data file. Copyright: © 2018 Abdulla Mohammed WS et al. 2018 Data associated with the article are available under the terms of the Creative Commons Zero "No rights reserved" data waiver (CC0 1.0 Public domain dedication).

## Discussion

Chloroquine has been widely used in many malaria endemic regions of many sub-Sahara African regions for its cost benefit and cost effective effects; and has therefore been considered as the golden choice in the treatment of malaria ^10^. In the current study, Chloroquine resistance showed an increase to 80% from the previously documented figure of 72.7% in 2007 in Sudan ^8^. This evidence differs from that documented from surveys in Malawi, Tanzania and Mozambique as in such regions there has been a decreasing trend of Chloroquine resistance prior to withdrawal of Chloroquine from the treatment guidelines ^11^.

In Malawi, resistant strains of *P. falciparum (Pfcrt*- T76) had declined from >t;85% to 0% within 13 years following Chloroquine withdrawal from treatment guidelines. In Tanzania *P. falciparum* resistance reduced from >80% to <10% in ten years, and in Mozambique statistics showed a decline of Chloroquine resistance from >95% to 20% within five years of Chloroquine withdrawal ^11^. However, studies from Kenya indicated a slow decline in Chloroquine resistant *P. falciparum* from 95% to 60% between 1993 to 2006 following malaria policy changes ^12^. The results of the present study are similar to studies from Uganda as the frequency of *Pfcrt-* T76 was between 100 and 98.7% in 2008, about eight years post-Chloroquine replacement ^11^.

In present study, the small sample size represents a limitation and the final result shows obvious persistence of *P. falciparum* chloroquine resistant strains in endemic areas where Chloroquine use was continued following the World Health Organization's declaration of sidelining Chloroquine as a first treatment choice for malaria.

## Conclusions

*Pfcrt* K76T mutation still persists in Sudan, which makes it impossible for the reintroduction of Chloroquine as an antimalarial treatment choice on the current National clinical guidelines of Sudan. However for a better implementation of this policy further studies need to be conducted with a larger representative sample from all regions of Sudan as this was a primary survey that involved a narrow sample.

## Data availability

The data referenced by this article are under copyright with the following copyright statement: Copyright: © 2018 Abdulla Mohammed WS et al.

Data associated with the article are available under the terms of the Creative Commons Zero "No rights reserved" data waiver (CC0 1.0 Public domain dedication). 

**Dataset 1: Raw sequences for the 20 patients included in the present study.** Files included: sequence in pdf format, sequence for opening in FinchTV software (.ab1 file), and sequence for opening in Notepad (TXT file). DOI, 10.5256/f1000research.13273.d192978 ^13^
