# Supplementary material for: Cross sectional study to determine chloroquine resistance among Plasmodium falciparum clinical isolates from Khartoum, Sudan
Source: F1000Res. 2018 Feb 20;7:208. [Version 1] doi: 10.12688/f1000research.13273.1 (PMC5958312; doi:10.12688/f1000research.13273.1)

File: crtd20_crtd1.ab1 *Run Ended: 2016/6/14 1:11:38* *Signal G:1989 A:2653 C:1076 T:4016*

Sample: crtd20_crtd1 *Lane: 33* *Base spacing: 14.30472* *183 bases in 2243 scans*

10 20 30 40 50 60 70 80 90 100 110 120

CAAA TA C G ATA TAT T T T TAT T TATAT T T TAA GTAT TAT T TAT T TAAGT GTAT GT GTAAT T AATACAAT T T T T GCTAAAAGAACT T TAAACAAAAT T G GTAACTATAGT T T T GTAACAT C CG

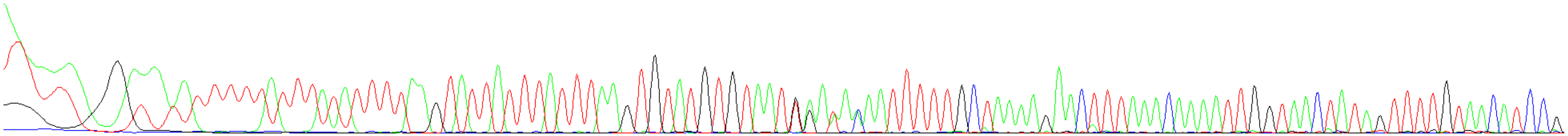

130 140 150 160 170 180

ACCAAAATTGGTAACTATATTTTTGTAACATCCGAAAAAAAATAATAAATA AATTTGTGT GT

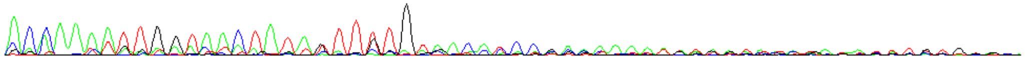

Supplement: Raw sequences for the 20 patients included in the present study — Files included: sequence in pdf format, sequence for opening in FinchTV software (.ab1 file), and sequence for opening in Notepad (TXT file). [file f1000research-7-14400-s0000.tgz › 8686ace3-bf9f-4a84-8ddc-77b50209d946_Data.zip!/Data/crtd 20/crtd20_crtd1.pdf]
