# Supplementary material for: Cross sectional study to determine chloroquine resistance among Plasmodium falciparum clinical isolates from Khartoum, Sudan
Source: F1000Res. 2018 Feb 20;7:208. [Version 1] doi: 10.12688/f1000research.13273.1 (PMC5958312; doi:10.12688/f1000research.13273.1)

File: crtd4_crtd1.ab1 *Run Ended: 2016/6/14 1:11:38* *Signal G:3522 A:5981 C:1169 T:9237*

Sample: crtd4_crtd1 *Lane: 17* *Base spacing: -15.016356* *114 bases in 1387 scans* *Page 1 of 1*

10 20 30 40 50 60 70 80 90 100 110

CAAAATACGATATATTTTTATTTATATTTTAAGTATTATTTATTTAAGTGTATGTGTAATTAATAAAATTTTTGCTAAAAGAACTTTAAACAAAATTGGTAACTATAGTTTTGT

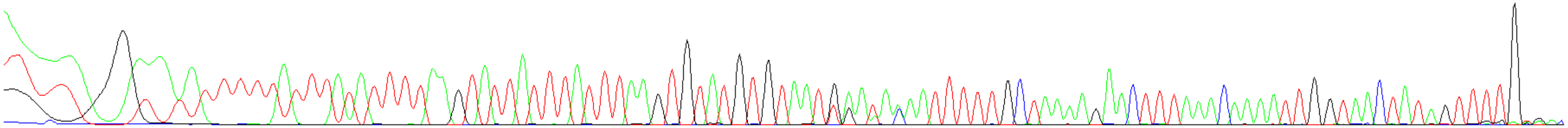

Supplement: Raw sequences for the 20 patients included in the present study — Files included: sequence in pdf format, sequence for opening in FinchTV software (.ab1 file), and sequence for opening in Notepad (TXT file). [file f1000research-7-14400-s0000.tgz › 8686ace3-bf9f-4a84-8ddc-77b50209d946_Data.zip!/Data/crtd 4/crtd4_crtd1.pdf]
