# Supplementary material for: Cross sectional study to determine chloroquine resistance among Plasmodium falciparum clinical isolates from Khartoum, Sudan
Source: F1000Res. 2018 Feb 20;7:208. [Version 1] doi: 10.12688/f1000research.13273.1 (PMC5958312; doi:10.12688/f1000research.13273.1)
Supplement: Peer review history

# Peer review: Referee response for version 1

**DOI:** 10.5256/f1000research.14400.r31878

**Published:** 2018-05-16

**Authors:** Gabriel N. Magoma

**Affiliations:** 1 Institute for Basic Sciences, Technology and Innovation (PAUSTI), Pan African University, Juja, Kenya

The rationale for the research on the determination of prevalence chloroquine resistance among clinical isolates is generally sound given the significance of the Malaria disease.

 The statement that there chloroquine has been in used despite the ban is not clearly articulated. The argument supporting the reintroduction of chloroquine is not well articulated. The sample size is also quite small and the selection criteria of study participants is not well supported. There is an implication of inclusion of children statement that there were 13 women and 7 men contradict the inclusion of children.

 The methods are not sufficiently detailed since the gel photos of the amplification products are missing and there is lack of explanation as to how the products were treated before sequencing. If this are articulated it is possible to ensure that the methods are fully reproducible.

 The analysis of the presence of the mutation, the interpretation and subsequent conclusions are generally adequate.

I have read this submission. I believe that I have an appropriate level of expertise to confirm that it is of an acceptable scientific standard.

# Peer review: Referee response for version 1

**DOI:** 10.5256/f1000research.14400.r33752

**Published:** 2018-05-08

**Authors:** Satish K. Dhingra

**Affiliations:** 1 Department of Microbiology and Immunology, Columbia University Medical Center, New York City, NY, USA

1. The premise of this study uses PfCRT K76T as a molecular maker of chloroquine resistance. However, a recent study from French Guiana in South America has shown that despite the presence of PfCRT K76T parasites can be sensitive to chloroquine (Pelleau *et al*. 2015, *PNAS*). So stating that parasite population in Sudan are clinically resistance to CQ based only upon the status of position 76 is incorrect. The authors need to sequence full length *pfcrt* in order to draw that conclusion.

 2. Overall the sample size is small and not very diverse.

 3. Authors fail to cite key publications, as an example Fidock *et al*. 2000, *Mol Cell*. 

 4. First sentence, second paragraph in discussion is missing a citation.

 5. The authors need to also elaborate of the prevalence of *P. vivax *in the area and whether reintroduction of CQ is for targeting this species and how this can impact the population structure of the *P. falciparum.*

I have read this submission. I believe that I have an appropriate level of expertise to confirm that it is of an acceptable scientific standard, however I have significant reservations, as outlined above.

# Peer review: Referee response for version 1

**DOI:** 10.5256/f1000research.14400.r31876

**Published:** 2018-04-03

**Authors:** Jagadish Mahanta

**Affiliations:** 1 Regional Medical Research Centre, Indian Council of Medical Research (ICMR), Dibrugarh, India

1. The concept is old and methodology is well established, however geography specific information still has the relevance and the results of such studies have policy implications in the programme. In this context, the investigators should give elaborate justification for revisiting resistance pattern at molecular level by including the clinical experience of physicians or in-vitro drug sensitivity using CHQ across the country.
2. As the studies of such nature have geographical relevance, the coverage should have been wide rather than limiting in a small area.
3. As stated in the text, drug policy has been changed since 2004 and chloroquine is no longer in use, therefore sampling should have been from different areas of the country and detail description of the patient’s therapeutic history, residential status and travel detail prior to infection should have been included.
4. Investigator should elaborate on site selection, patient selection and randomization detail during sampling.

I have read this submission. I believe that I have an appropriate level of expertise to confirm that it is of an acceptable scientific standard, however I have significant reservations, as outlined above.
